# Mobility-oriented measurements of people’s exposure to outdoor artificial light at night (ALAN) and the uncertain geographic context problem (UGCoP)

**DOI:** 10.1371/journal.pone.0298869

**Published:** 2024-04-26

**Authors:** Yang Liu, Mei-Po Kwan

**Affiliations:** 1 Department of Geography and Resource Management, The Chinese University of Hong Kong, Hong Kong, Special Administrative Region of China; 2 Institute of Space and Earth Information Science, The Chinese University of Hong Kong, Hong Kong, Special Administrative Region of China; 3 Institute of Future Cities, The Chinese University of Hong Kong, Hong Kong, Special Administrative Region of China; ULAB: University of Liberal Arts Bangladesh, BANGLADESH

## Abstract

Advanced nighttime light (NTL) remote sensing techniques enable the large-scope epidemiological investigations of people’s exposure to outdoor artificial light at night (ALAN) and its health effects. However, multiple uncertainties remain in the measurements of people’s exposure to outdoor ALAN, including the representations of outdoor ALAN, the contextual settings of exposure measurements, and measurement approaches. Non-exposed but included outdoor ALAN and causally irrelevant outdoor ALAN may manifest as contextual errors, and these uncertain contextual errors may lead to biased measurements and erroneous interpretations when modeling people’s health outcomes. In this study, we systematically investigated outdoor ALAN exposure measurements in different geographic contexts using either residence-based or mobility-oriented measurements, different spatial scales, and multiple NTL data sources. Based on the GPS data collected from 208 participants in Hong Kong, outdoor ALAN exposures were measured from NTL imagery at 10 m, 130 m, and 500 m spatial resolutions using *in-situ* methods or 100 m, 300 m, and 500 m buffer zone averaging. Descriptive analysis, multiple t-tests, and logistic regression were employed to examine the differences between outdoor ALAN exposure measurements using various contextual settings and their effects on modeling people’s overall health. Our results confirmed that different contextual settings may lead to significantly different outdoor ALAN exposure measurements. Our results also confirmed that contextual errors may lead to erroneous conclusions when using improper contextual settings to model people’s overall health. Consequentially, we suggest measuring people’s exposure to outdoor ALAN using the mobility-oriented approach, NTL representation with the high spatial resolution, and a very small buffer zone as a contextual unit to derive outdoor ALAN exposure. This study articulates essential methodological issues induced by uncertainties in outdoor ALAN exposure measurements and can provide essential implications and suggestions for a broad scope of studies that need accurate outdoor ALAN exposure measurements.

## Introduction

People’s exposure to artificial light at night (ALAN) has been found to have complicated associations with their physical and mental health [1]. Extra light at night may disturb human circadian rhythms [2], which leads to extra stress on the human neuroendocrine system and sleep, and people’s chronic exposure to ALAN has been observed to have significant associations with breast cancer [3,4], obesity [5], and sleep disorders [6]. However, acute exposure to ALAN may not directly lead to adverse health effects on humans since the human body has very strong resilience [1]. Meanwhile, outdoor ALAN may provide people with bright space for nighttime activities and remove some environmental barriers for physical exercises [7]. Adequate outdoor brightness may also reduce residents’ perception of fear and increase their perception of neighborhood safety [8], and the beautiful nightscape may promote people’s mental health and life satisfaction [9].

People’s exposure to ALAN is the most essential link between ALAN and human health. Conventional studies used laboratory experiments with well-controlled indoor illumination to research human photobiological reactions to ALAN (e.g., [10–13]). These laboratory experiments well explored the fundamental mechanisms of ALAN’s health effects. However, the well-controlled laboratory settings of indoor illumination are very difficult to replicate in people’s everyday life, which may undermine the practical value of these laboratory-based studies. In recent years, epidemiological investigations of people’s exposure to ALAN in real life become prevalent for examining ALAN’s health effects (e.g., [14–16]). Significant associations with human health outcomes have been observed for both indoor ALAN exposure and outdoor ALAN exposure [1]. The development of remote sensing techniques like nighttime light (NTL) imaging further enabled the large-scope epidemiological investigations of very huge cohorts and populations [17].

Proper measurements of people’s exposure to environmental factors require proper representations of environmental settings, proper representations of human behaviors, and the proper contextual units to confine measurements [18]. One essential issue of current epidemiological investigations of outdoor ALAN’s health effects is ignoring people’s nighttime mobility. These studies confined people’s ALAN exposure to the residential context (e.g., a neighborhood or an administrative area [19–24]) and used outdoor ALAN statistics within the residential contexts (e.g., average value) to assess their exposure to outdoor ALAN. In this way, the spatial variation in residential outdoor ALAN is considered the variation of people’s exposure to outdoor ALAN. These measurements are thus residence-based measurements (RBM). However, people may be exposed to different intensities of outdoor ALAN as they visit different locations because the spatial distribution of outdoor ALAN is not even. Further, people’s nighttime activity spaces may still be considerable even if they are significantly smaller than people’s daytime activity spaces. Ignoring people’s nighttime mobility may thus lead to significant contextual errors in the measured exposure to outdoor ALAN and misleading conclusions, manifesting the uncertain geographic context problem (UGCoP) [25]. Hence, we argue that it is necessary to incorporate people’s nighttime mobility and develop mobility-oriented measurements (MOM) to study ALAN’s health effects, especially for individual-level epidemiological investigations and analyses of causally relevant pathways.

**Fig 1:**
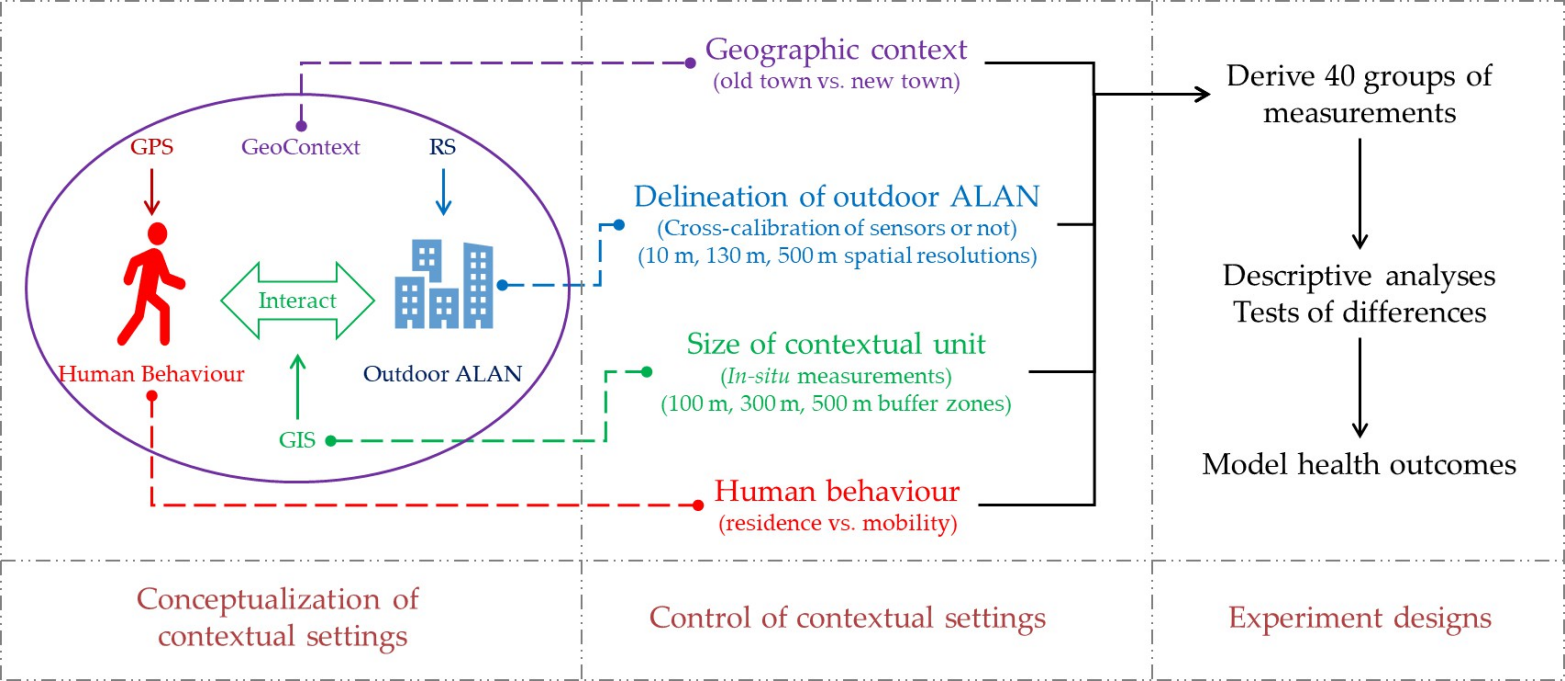
The conceptual framework of the systematic investigation of the UGCoP in measuring people’s exposure to outdoor ALAN.

MOM may effectively mitigate the contextual errors in outdoor ALAN exposure measurements, and the current development and prevalence of portable GPS devices further enable the precise delineation of people’s activity-travel trajectories with high temporal resolution and adequate spatial accuracy. However, other factors may also induce measurement uncertainties when measuring people’s exposure to outdoor ALAN, such as the spatial resolution of outdoor ALAN representation [26]. A systematic investigation of these factors and uncertainties is still absent, which makes it difficult for researchers to find proper and causally relevant contextual settings when measuring people’s exposure to outdoor ALAN. To fill this research gap, this study conducted a systematic investigation of the contextual settings for outdoor ALAN exposure measurements. We conceptualized the possible measurement uncertainties induced by the potentially important factors from four controls of contextual settings (Fig 1 presents the conceptual framework of our systematic investigation). In the following sections, we first introduce our study area and data. We then detail our methods and 40 groups of designated outdoor ALAN exposure measurements using various contextual settings. The disparities between these measurements are summarized in the Results Section. The implications and limitations of this study are also highlighted in the Discussions Section. Finally, the main conclusions are drawn from our results and discussions.

## Methods and materials

### Study area

**Fig 2:**
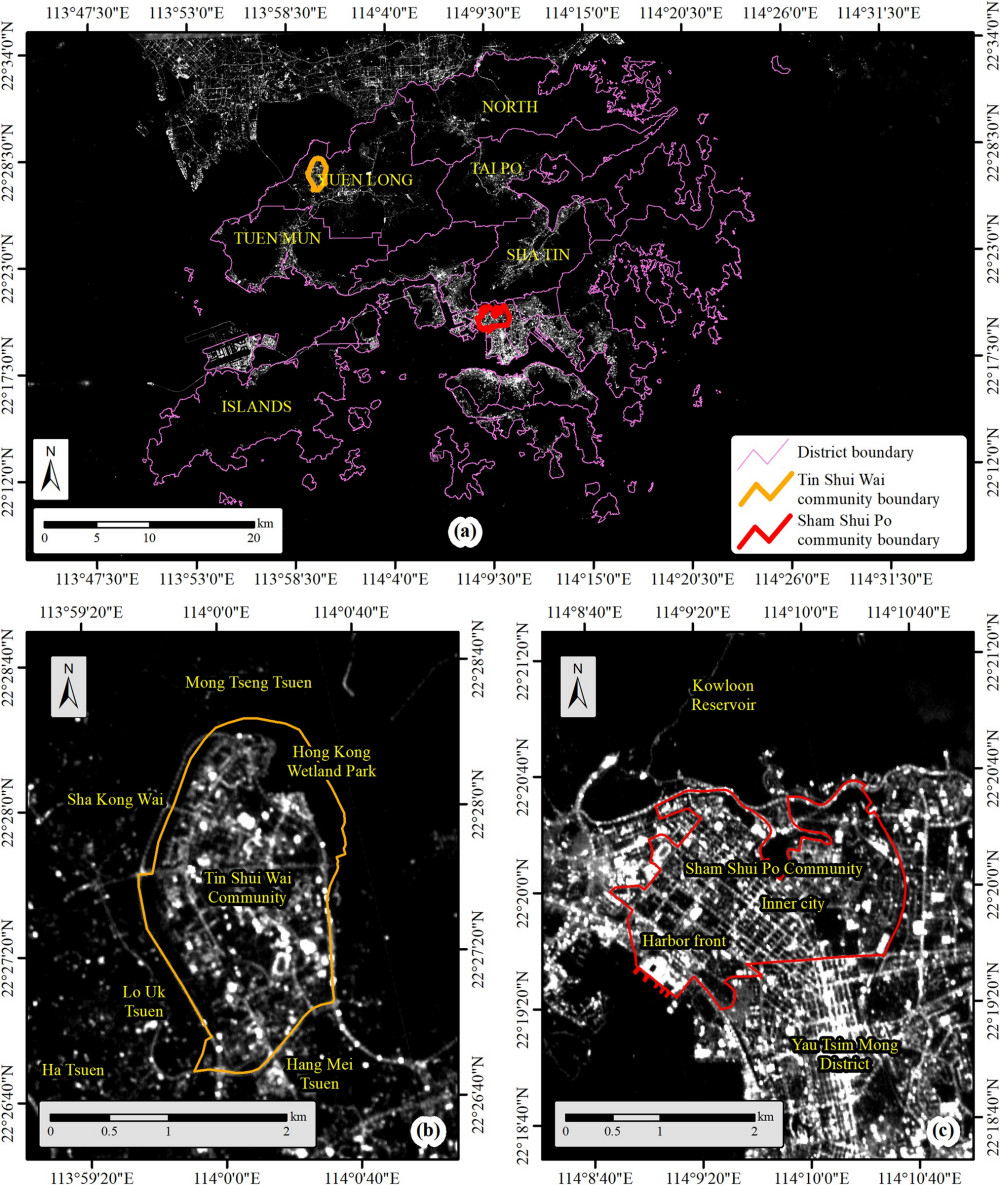
The nightscapes of SSP and TSW. (a). The locations of SSP and TSW in Hong Kong, (b). the nightscape of TSW, and (c). the nightscape of SSP. Background is from the 10-m spatial resolution SDGSAT-1 Glimmer imagery. The base map is reprinted from SDGSAT-1 Open Science Program under a CC BY 4.0 license, with permission from SDGSAT-1 Chief Scientist Office, original copyright 2022.

We choose Hong Kong as our study area, which is one of the world’s most urbanized and densely populated cities with well-developed nightscapes. Particularly, we choose two representative communities in Hong Kong to conduct our field survey, one is the Sham Shui Po (SSP) community and the other is the Tin Shui Wai (TSW) community (Fig 2). The blocks of the SSP community included in our study have an area of about 5.35 km^2^ and a population of about 300,000 by 2018 [27]. SSP is an old town developed in the early stage of Hong Kong. Correspondingly, SSP generally has low and crowded buildings enclosed by bright streets at night and a nice nightscape (Fig 2C). The average luminosity in SSP is about 325.32 *nW*∙*cm*^−2^∙*sr*^−1^ (derived from calibrated SDGSAT-1 Glimmer imagery). The TSW community has an area of about 4.32 km^2^ and a similar residential population of about 300,000 by 2018 [27]. Different from SSP, TSW is a new town developed in the 1980s. The urban planning of TSW contains more open areas and the community is enclosed by more rural geographic settings, such as the Hong Kong Wetland Park and Hong Kong’s fish farm regions. The nightscape in TSW is less bright than that in SSP (Fig 2B) and the average luminosity in TSW is about 210.78 *nW*∙*cm*^−2^∙*sr*^−1^.

### Remote sensing data collection

We collected multiple sets of NTL remote sensing image data from three data sources to investigate the measurement uncertainties induced by nightscape delineation using different sensor configurations. The first NTL data source is the Suomi National Polar Partnership/Visible Infrared Imaging Radiometer Suite (SNPP/VIIRS) Day Night Band (DNB) daily mosaic imagery at the nominal 500 m spatial resolution [28], the second is the Luojia1-01 CMOS Panchromatic imagery at the nominal 130 m spatial resolution [29], and the last is the Sustainable Development Science Satellite 1 (SDGSAT-1) Glimmer imagery at the 10 m spatial resolution [30]. Our data collection is currently the most comprehensive NTL data collection that can be used to measure people’s exposure to outdoor ALAN. More details about the data’s attributes are shown in Table 1.

**Table 1:** The sensor configurations of the NTL remote sensing data in this study.

Data source	Acquisition date	Imaging time (local time)	Spatial resolution	Spectral range	Bandwidth	Cloud occlusion
SDGSAT-1	12/7/2022	21:45:34.8	10 m	444–910 nm	466 nm	None
Luojia1-01	24/11/2018	22:46:52.2	130 m ^b^	460–980 nm	520 nm	Partially ^c^
SNPP/VIIRS	8/12/2022	Daily mosaic ^a^	500 m ^b^	500–900 nm	400 nm	None

^a^ VIIRS image used in this study is a daily mosaic product, whose concrete imaging time is not available.

^b^ Nominal spatial resolution according to the official introduction, actual spatial resolution may be slightly better in Hong Kong.

^c^ According to the 3-day daytime observations from PlanetScope images, the Luojia1-01 NTL image may be partially cloudy. However, the cross-validation indicates it still can be used.

SNPP/VIIRS images were collected from the National Oceanic and Atmospheric Administration (NOAA) National Centers for Environmental Information (https://ngdc.noaa.gov/eog/viirs/download_ut_mos.html), Luojia1-01 images were collected from the Hubei Data and Application Center (http://datasearch.hbeos.org.cn), and SDGSAT-1 images were collected from the International Research Center of Big Data for Sustainable Development Goals (http://www.sdgsat.ac.cn/). SNPP/VIIRS image product is pre-calibrated in the unit of *nW*∙*cm*^−2^∙*sr*^−1^ [31]. Luojia1-01 images are calibrated using the following equation [32,33]:

$$L_{Luojia1-01}=\frac{{DN}^{1.5}}{10^{10}},$$ (1)

where *L*_*Luojia*1−01_ is the luminosity in the unit of *W*∙*m*^−2^∙*sr*^−1^∙*μm*^−1^, and *DN* is the digital number value on the original uncalibrated image. SDGSAT-1 images are calibrated using the equation [34]:

$$L_{SDGSAT-1}=DN*Gain+Bias,$$ (2)

where *L*_*SDGSAT*−1_ is the luminosity in the unit of *W*∙*m*^−2^∙*sr*^−1^∙*μm*^−1^, *DN* is the digital number value on the original uncalibrated image, and *Gain* and *Bias* are the linear calibration coefficients collected from the metadata of the product. To be consistent with SNPP/VIIRS and to have convenient numbers, the unit of luminosity on Luojia1-01 and SDGSAT-1 images are both converted to *nW*∙*cm*^−2^∙*sr*^−1^.

All three sets of images are orthorectified and georeferenced to UTM Zone 49N coordinate system based on the WGS84 ellipsoid, and they all can fully cover the entire Hong Kong. The SNPP/VIIRS and SDGSAT-1 images both are cloud-free. While the Luojia1-01 image may be partially cloudy, the clouds do not occlude the main parts of Hong Kong and using it would not lead to significant biases in outdoor ALAN exposure measurements. The SNPP/VIIRS and SDGSAT-1 images are the most updated images that can match well with our field survey. The available Luojia1-01 image is earlier than our field survey. However, since the nightscape in Hong Kong does not significantly change in the short term, we assume that the slight mismatch of image acquisition time does not lead to significant biases in outdoor ALAN exposure measurements.

Due to the apparent disparities between sensor configurations (Table 1), the calibrated NTL images may still not yield equivalent luminosity for the proper comparison of outdoor ALAN exposure measurements. To mitigate the disparities induced by sensor configurations, we also employed multiple linear models for cross-calibration between different data sources. We used the luminosity on the SDGSAT-1 image as the reference, randomly collected about 300 samples (the maximum sample size that can be collected from SNPP/VIIRS image) in Hong Kong, and established two linear models to predict the equivalent luminosity of SDGSAT-1 images from either SNPP/VIIRS or Luojia1-01 images. The 1-km buffer zones and the mean values within buffer zones are used instead to mitigate the effects of different spatial resolutions. *R*^2^ is employed as the indicator for evaluating the goodness of cross-calibration between sensors.

### GPS-derived activity-travel trajectories and questionnaires

The data collected from participants contain confidential private information and the project was reviewed and approved by the *Survey and Behavioral Research Ethics (SBRE) Committee* of the Chinese University of Hong Kong (Reference No. SBRE-19-123 approved on 8 January 2020). Written informed consent was obtained from all subjects involved in the study before data were collected from them.

To precisely delineate participants’ nighttime activity space, we asked participants to record and submit data through an integrated individual environmental exposure assessment system (IEEAS) [35] consecutively for 7 days (5 weekdays and 2 weekends). The visited locations of the 7 survey days were collected from each participant’s GPS-equipped mobile phone and then assembled using the Kalman filter [36]. A time-series of sequentially visited locations (in longitude and latitude) of each participant was derived at the 1-minute temporal resolution to retrospectively delineate the activity-travel trajectory during the 7-day survey period [37].

We also collected participants’ socio-demographic attributes, home addresses, and overall health statuses through questionnaires. In the questionnaire, each participant was asked to rate his or her overall health status. The response is provided on a 6-point scale ranging from excellent to terrible. Due to the comparatively small sample size, the health status responses were dichotomized as a binary variable based on either an overall good health status (excellent, very good, and good) or an overall bad health status (bad, very bad, and terrible).

In total, our pilot field survey successfully recruited 222 participants from SSP and TSW using a stratified sampling method, which is part of a larger project. The socio-demographic characteristics of the participants were designed to be representative of the characteristics of each community. Multiple axes of the socio-demography were used for stratification, including age, gender, employment status, and monthly household income [27]. The field survey was carried out from March 21^st^, 2021, to September 12^th^, 2021. By excluding void responses and incomplete activity trajectories, the survey finally yielded valid data from 208 participants, including 104 in SSP and 104 in TSW, respectively. A range of socio-demographic statuses through multiple axes can be adequately covered in our recruited participants (Table 2).

**Table 2:** The socio-demographic profiles and self-reported overall health status in SSP/TSW.

Variable		SSP	TSW
Gender	Male	44 (42.3%)	48 (46.2%)
	Female	60 (57.7%)	56 (53.8%)
Age	18–24	16 (15.4%)	21 (20.2%)
	25–44	52 (50.0%)	51 (49.0%)
	45–64	36 (34.6%)	32 (30.8%)
Monthly household income ^a^	Low	47 (45.2%)	27 (26.0%)
	Middle	32 (30.8%)	48 (46.2%)
	High	25 (24.0%)	29 (27.8%)
Education level ^b^	Low	37 (35.6%)	36 (34.6%)
	Middle	55 (52.9%)	56 (53.8%)
	High	12 (11.5%)	12 (11.5%)
Marital status ^c^	Single	53 (51.0%)	58 (55.8%)
	Married	40 (38.5%)	35 (33.7%)
	Others	11 (10.6%)	11 (10.6%)
Overall health status	Excellent/very good/good	91 (87.5%)	93 (89.4%)
	Bad/very bad/terrible	13 (12.5%)	11 (10.6%)
**Total**		104 (100.0%)	104 (100.0%)

^a^ Monthly household income: The low-income group has an income of less than 20,000 Hong Kong dollars (HKD), the middle-income group has an income of 20,000 ~ 39,999 HKD, and the high-income group has an income of 40,000 HKD or above.

^b^ Education level: The low group graduated from middle school or lower, the middle group is with a bachelor’s degree or certification, and the high group is with a master’s degree or higher.

^c^ Other marital statuses include those divorced and widowed.

### Measuring participants’ exposure to outdoor ALAN

We defined two types of outdoor ALAN exposure measurements (i.e., *in-situ* measurements and buffer zone average measurements) for residence-based measurements (RBM) and mobility-oriented measurements (MOM), respectively. For the RBM, the *in-situ* outdoor ALAN exposure measurement is the pixel value on the NTL image of a participant’s home location:

$$E_{ALAN\_RBM\_in-situ}={NTL}_{p}(x_{h},y_{h}),$$ (3)

where *NTL*_*p*_(*x*_*h*_, *y*_*h*_) is the NTL image pixel that contains a participant’s home location (*x*_*h*_, *y*_*h*_), *p* is the spatial resolution of the NTL images, and *p* = 10 m, 130 m, and 500 m, respectively. The first type of measurement is applied to all three sets of NTL images. However, since the spatial resolution of SDGSAT-1 is high (10 m), *in-situ* measurements may not adequately capture all causally relevant outdoor ALAN exposure. We also derived the second type of outdoor ALAN exposure measurement using the buffer zone average of NTL pixel values:

$$E_{ALAN\_RBM\_buf}=\frac{\int_{buf_{r}}NTL_{p}(x,y)da}{\int_{buf_{r}}da},$$ (4)

where *buf*_*r*_ is the buffer zone around a participant’s home location with radius *r*, and *r* = 100 m, 300 m, and 500 m, respectively. *NTL*_*p*_(*x*, *y*) is the SDGSAT-1 NTL image pixel that is within the designated buffer zone, and *p* = 10 m for the SDGSAT-1 NTL image. *da* is the pixel’s areal size. The buffer zone average measurements are not applied to SNPP/VIIRS and Luojia1-01 NTL images since their coarse spatial resolutions are not adequate to match the generally used buffer zones.

For the MOM of outdoor ALAN exposure given an activity-travel trajectory in the form of a series of visited locations *P*(*x*_*i*_, *y*_*i*_, *t*_*i*_), *i* = 1, 2, 3…, the total exposure is defined as an accumulation of a series of momentary exposure to outdoor ALAN at each location and weighted by the duration of exposure at that location [37]. Particularly, for the *in-situ* measurement:

$$E_{ALAN\_MOM\_in-situ}=\sum {NTL}_{p}(x_{i},y_{i}){WT}_{i},$$ (5)

where *NTL*_*p*_(*x*_*i*_, *y*_*i*_) is the *in-situ* momentary exposure to outdoor ALAN at the i-th visited location (*x*_*i*_, *y*_*i*_), and the temporal weight *WT*_*i*_ at the i-th moment *t*_*i*_ within the duration (*D*) of the time-series is:

$${WT}_{i}=\frac{t_{i+1}-t_{i}}{D}.$$ (6)

On the other hand, for the buffer zone average measurement:

$$E_{ALAN\_MOM\_buf}=\sum {{WS}_{i}WT}_{i},$$ (7)

and the momentary buffer zone average outdoor ALAN exposure *WS*_*i*_ is:

$${WS}_{i}=\frac{\int_{{buf}_{r}}{NTL}_{p}(x_{i},y_{i})da}{\int_{{buf}_{r}}da}.$$ (8)

The MOM of people’s exposure to outdoor ALAN should be confined to nighttime by definition. We used the sunset and sunrise moments as the critical moments to define nighttime: the phase after sunset and before sunrise is defined as the nighttime and the rest is defined as the daytime [38]. The daytime momentary exposures to outdoor ALAN are assigned a value of 0. An R package is employed to facilitate the calculation of sunrise and sunset moments (https://CRAN.R-project.org/package=suncalc, accessed on 10 January 2023).

### Statistical analysis

**Fig 3:**
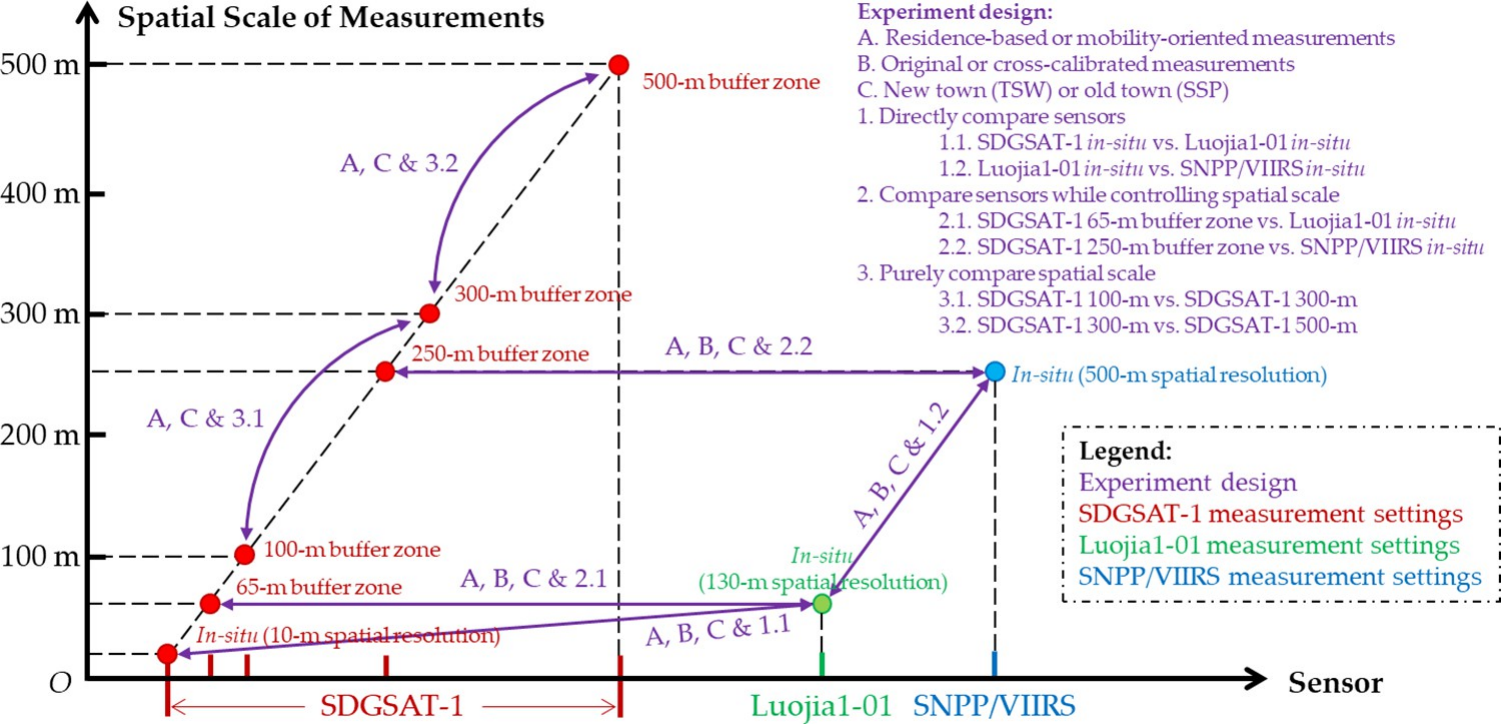
The experimental design to systematically investigate the disparities between outdoor ALAN measurements.

We employed multiple paired sample t-tests and Welch two-sample t-tests to test the disparities between the measured outdoor ALAN exposures. Fig 3 gives an illustration of our systematic investigation of the disparities between different contextual settings. We implemented multiple groups of comparisons: the first group tested the disparities induced by NTL images using *in-situ* measurements. To mitigate the disparities induced by the vastly different spatial resolutions, we also derived the 65-m buffer zone average measurements from SDGSAT-1 image to match the 130-m spatial resolution Luojia1-01 *in-situ* measurements, and the 250-m buffer zone average measurements from SDGSAT-1 image to match the 500-m spatial resolution SNPP/VIIRS *in-situ* measurements. Finally, the third group purely compared different buffer zone radiuses using SDGSAT-1 measurements. In each group of measurements, we first tested the disparities between RBM and MOM of participants’ exposure to outdoor ALAN. We then compared the measurements derived from original NTL image values and the cross-calibrated NTL image values. Finally, we compared the measurements from different geographic contexts. In total, we derived 40 groups of outdoor ALAN exposure measurements using different contextual settings and systematically investigated the disparities induced by sensor configurations and spatial resolutions (SNPP/VIIRS/500 m, Luojia1-01/130 m, and SDGSAT-1/10m), buffer zone radiuses (100 m, 300 m, and 500 m), cross-calibration or not, measurement approaches (RBM vs. MOM, and *in-situ* vs. buffer zone average), and geographic contexts (SSP/old town vs. TSW/new town).

The contextual errors induced by improper contextual settings when measuring people’s exposure to outdoor ALAN may lead to misleading results when analyzing the effects of outdoor ALAN on human health. To articulate this issue, 40 binary logistic regression models were estimated to model participants’ self-reported overall health outcomes (the Logit values indicate the probability of a participant being healthy, which is the logarithmic value of the ratio of the probability of being healthy over the probability of being not healthy) using each measured exposure to outdoor ALAN as a predictor, respectively. Several socio-demographic variables are also incorporated into these models to control for the effects of possible confounders, including age, gender, educational level, marital status, and socio-economic status [37,39,40]. The effect size of each measured exposure to outdoor ALAN and the corresponding *p*-value are used to discuss the robustness of the measured exposure to outdoor ALAN across the gradients of contextual settings.

## Results

### The measured exposure to outdoor ALAN

**Fig 4:**
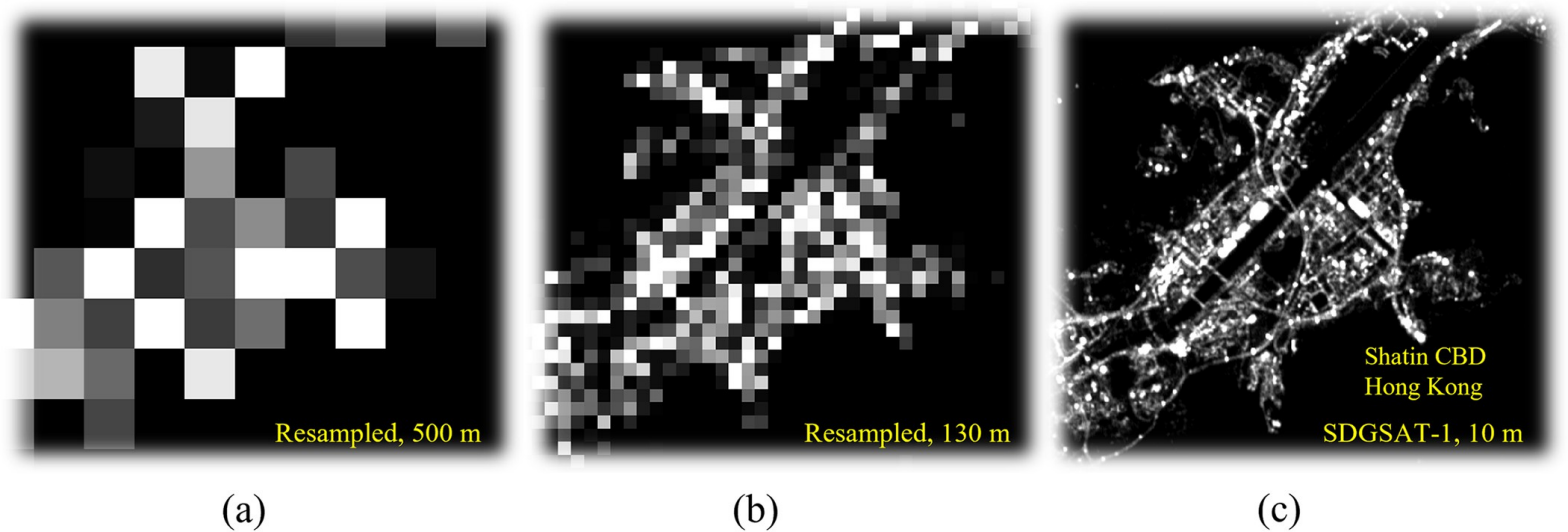
The disparities induced by the spatial resolutions of remote sensing data, using the nightscape in Sha Tin CBD as an example. (a) The resampled SDGSAT-1 imagery with 500-m spatial resolution, equivalent to the SNPP/VIIRS DNB imagery, (b) the resample SDGSAT-1 imagery with 130-m spatial resolution, equivalent to the Luojia1-01 Panchromatic imagery, and (c) the original SDGSAT-1 Glimmer imagery with 10-m spatial resolution. The base map is reprinted from SDGSAT-1 Open Science Program under a CC BY 4.0 license, with permission from SDGSAT-1 Chief Scientist Office, original copyright 2022.

The nightscapes of Hong Kong were successfully delineated using the three sets of NTL images employed in this study, and the cross-calibration has been successfully applied to them through linear models with adequately high *R*^2^ (Table 3). The high *R*^2^ values indicate that the delineated nightscapes from different remote sensing satellites can be consistent with each other. However, the coefficients of the cross-calibration are apparently different from 1, and the luminosity derived from the sensor with a lower spatial resolution seems to be underestimated. Hong Kong government gradually replaced the Hong Kong street lights with LED lights since 2017 [41], and LED emission has one blue peak centered around about 450 nm (420 nm– 480 nm) [42]. SDGSAT-1’s sensor fully intakes this peak, Luojia1-01’s sensor partially intakes this peak, while SNPP/VIIRS’s sensor fully excludes this peak (Table 1 spectral ranges of the sensors). The disparity in energy intake may be the primary reason for the underestimation of the sensor with a lower spatial resolution. This disparity also emphasizes that cross-calibration between NTL images may be necessary to mitigate the measurement uncertainties induced by the sensor spectral configurations. On the other hand, the spatial resolution itself may also be an essential source that leads to measurement uncertainties (Fig 4). Because urban nightscapes have drastic changes across space, a coarse spatial resolution (SNPP/VIIRS, 500 m) may not adequately and precisely capture people’s exposure to outdoor ALAN within small nighttime activity spaces.

**Table 3:** The cross-calibration between different remote sensing NTL data.

Reference	Predictor	Equation^a^	*R* ^2^	Sample Size
SDGSAT-1	Luojia1-01	$L_{SDGSAT-1}=1.47*L_{Luojia1-01}+23.14$	0.925	294
SDGSAT-1	SNPP/VIIRS	$L_{SDGSAT-1}=2.20*L_{SNPP/VIIRS}+11.71$	0.805	294
Luojia1-01	SNPP/VIIRS	$L_{Luojia1-01}=1.46*L_{SNPP/VIIRS}-6.93$	0.857	294

^a^ Unit of luminosity (*L*): *nW*∙*cm*^−2^∙*sr*^−1^.

**Fig 5:**
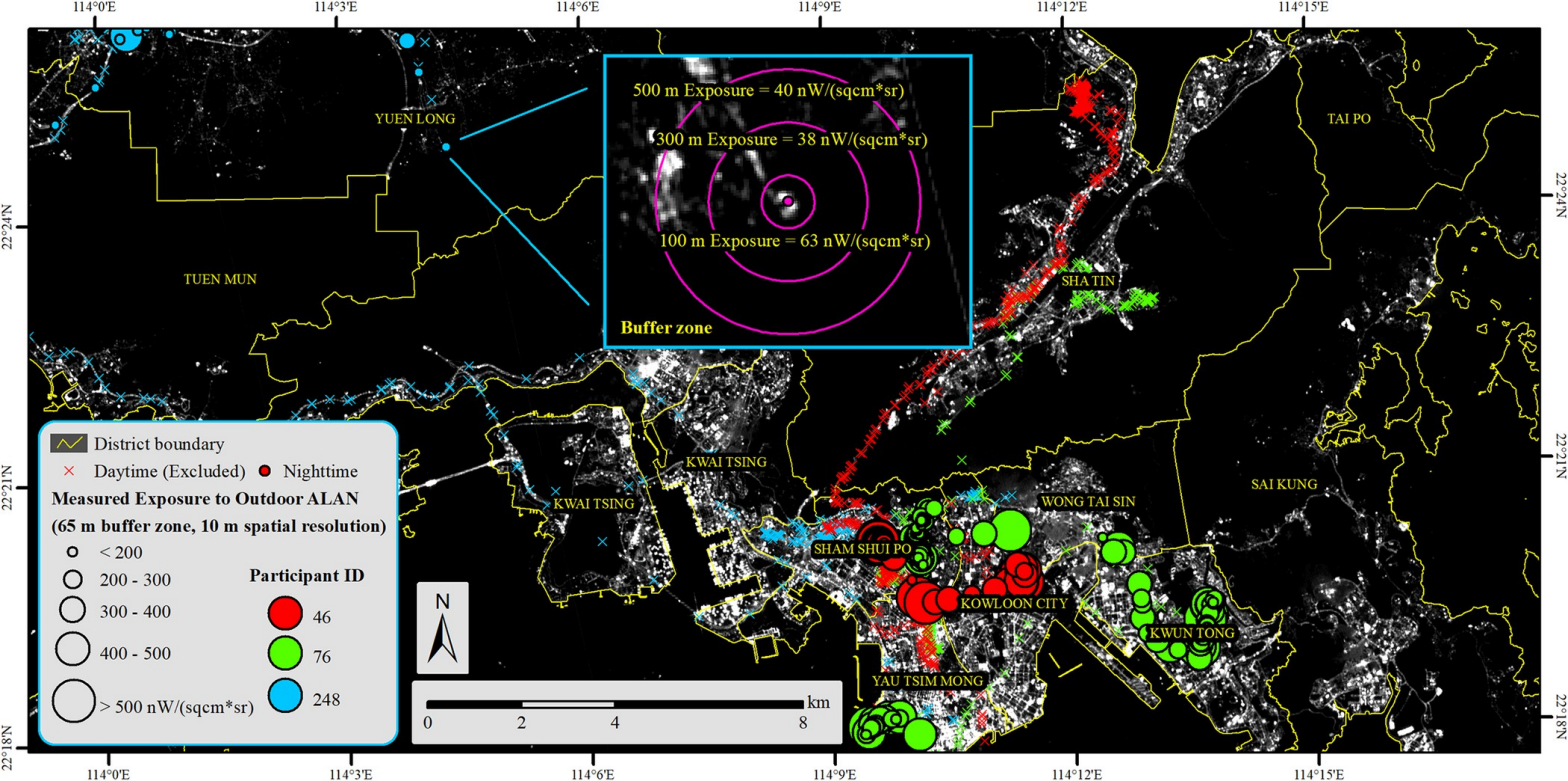
The spatial variation of exposure to outdoor ALAN. Apparent spatial variation can be observed along a participant’s nighttime activity-travel trajectory. The base map is reprinted from SDGSAT-1 Open Science Program under a CC BY 4.0 license, with permission from SDGSAT-1 Chief Scientist Office, original copyright 2022.

Ignoring people’s nighttime mobility is another essential source of contextual errors when measuring people’s exposure to outdoor ALAN. Fig 5 provides an example to illustrate participants’ nighttime activity spaces and the corresponding momentary outdoor ALAN exposure measurements. People may have lower mobility during the nighttime than that during the daytime, but their activity spaces may still be significantly larger than the conventionally assumed 500-m buffer zone around home locations. Meanwhile, people’s exposure to outdoor ALAN may have drastic changes in different visited locations, while ignoring people’s nighttime mobility may exclude a large amount of actual exposure to outdoor ALAN along their activity-travel trajectories and then may manifest the UGCoP.

The buffer zone radius may also be an influential factor in the measurements of people’s exposure to outdoor ALAN. Because the *in-situ* measurements may not adequately capture all the outdoor ALAN exposure on the fine-grained NTL imagery (e.g., SDGSAT-1 Glimmer imagery), a buffer zone may be necessary to include all the causally relevant outdoor ALAN exposure. However, since the nightscape in the urban area changes drastically across space, different buffer zone sizes may yield significantly different measurement results (Fig 5). A too-large buffer zone may either include much outdoor ALAN that a person does not get exposed to or underestimate the exposure intensity through the average effect of non-essential dark areas, which also manifests the UGCoP.

### The disparities induced by contextual settings

**Fig 6:**
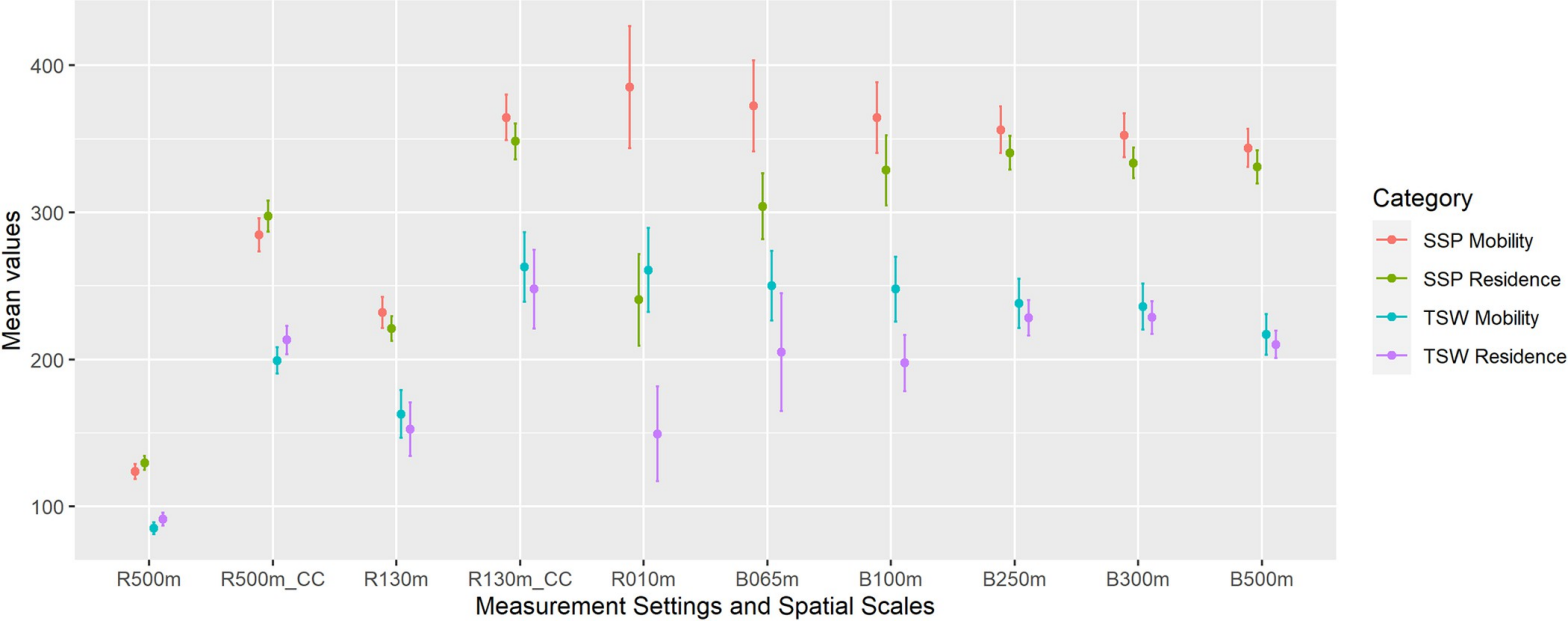
The mean values of measured exposure to outdoor ALAN using the designated 40 groups of contextual settings, bounded with 95% confidence intervals. r???m: *In-situ* measurements using the given spatial resolution; CC: Cross-calibrated; b???m: Zonal mean value using the given buffer radius. Unit of outdoor ALAN exposure mean values: *nW*∙*cm*^−2^∙*sr*^−1^.

Forty groups of outdoor ALAN exposure measurements were successfully derived using our SSP and TSW sample sets with different contextual settings (Fig 6). We also observed a range of significant disparities induced by different contextual settings (Tables 4–7). The primary disparities are between geographic contexts (Table 4). All SSP exposure measurements are significantly higher than TSW exposure measurements, which is consistent with the nightscape conditions within these two communities (Fig 2). Meanwhile, both coarser spatial resolutions and larger buffer zone radiuses may magnify the disparities between geographic contexts. The second group of significant disparities can be observed between the RBM and MOM (Table 5). Multiple significant disparities can be observed except for these measurements using larger buffer zones in TSW and the Luojia1-01 measurements. Most measurements using MOM are larger than those using RBM except for these SNPP/VIIRS measurements. Coarser spatial resolution tends to invert the disparities between RBM and MOM (disparities change from positive to negative), and a larger buffer zone tends to mitigate the disparities between RBM and MOM.

**Table 4:** Two-sample t-tests of differences between different geographic contexts.

Mobility-oriented measurements				Residence-based measurements			
Setting ^a^	D (SE)	*t*	*p*-value	Setting ^a^	D (SE)	*t*	*p*-value
M_r500m	38.77 (3.31)	11.701	<0.001**	R_r500m	38.19 (3.34)	11.441	<0.001**
M_r500m_CC	85.44 (7.30)	11.701	<0.001**	R_r500m_CC	84.15 (7.36)	11.441	<0.001**
M_r130m	69.13 (9.84)	7.024	<0.001**	R_r130m	68.30 (10.21)	6.691	<0.001**
M_r130m_CC	101.73 (14.48)	7.024	<0.001**	R_r130m_CC	100.51 (15.02)	6.691	<0.001**
M_r010m	124.32 (25.71)	4.835	<0.001**	R_r010m	91.10 (22.86)	3.985	<0.001**
M_b065m	122.50 (19.85)	6.170	<0.001**	R_b065m	99.19 (23.43)	4.234	<0.001**
M_b100m	116.52 (16.67)	6.989	<0.001**	R_b100m	131.09 (15.61)	8.398	<0.001**
M_b250m	118.00 (11.77)	10.029	<0.001**	R_b250m	112.26 (8.45)	13.281	<0.001**
M_b300m	116.48 (11.00)	10.589	<0.001**	R_b300m	105.06 (7.76)	13.537	<0.001**
M_b500m	126.81 (9.65)	13.139	<0.001**	R_b500m	120.78 (7.46)	16.195	<0.001**

Unit of the mean of difference (D) and standard error (SE): *nW*∙*cm*^−2^∙*sr*^−1^. (M: Mobility-oriented measurement, R: Residence-based measurement, CC: Cross-calibrated, r???m: *In-situ* measurement using the given spatial resolution, and b???m: Zonal mean value using the given buffer zone radius).

^a^ SSP measurements–TSW measurements. 104 samples in SSP and 104 samples in TSW, respectively.

**Significant different from 0 at the 0.01 level, 2-tailed tests and do not assume equal variances of two samples.

**Table 5:** Paired-sample t-tests of differences between mobility-oriented and residence-based measurements of people’s exposure to outdoor ALAN.

Pair	SSP (N = 104)			TSW (N = 104)		
	D (SE)	*t*	*p*-value	D (SE)	*t*	*p*-value
M_r500m - R_r500m	-5.75 (1.84)	-3.126	0.002**	-6.33 (1.74)	-3.633	0.000**
M_r500m_CC—R_r500m_CC	-12.67 (4.05)	-3.126	0.002**	-13.95 (3.84)	-3.633	0.000**
M_r130m - R_r130m	11.03 (6.51)	1.695	0.093	10.20 (8.59)	1.187	0.238
M_r130m_CC—R_r130m_CC	16.24 (9.58)	1.695	0.093	15.01 (12.65)	1.187	0.238
M_r010m - R_r010m	144.65 (21.36)	6.773	<0.001**	111.42 (21.16)	5.265	<0.001**
M_b065m - R_b065m	68.41 (13.41)	5.103	<0.001**	45.10 (20.23)	2.230	0.028*
M_b100m - R_b100m	35.79 (13.02)	2.750	0.007**	50.36 (10.60)	4.749	<0.001**
M_b250m - R_b250m	15.71 (7.17)	2.192	0.031*	9.97 (8.23)	1.211	0.229
M_b300m - R_b300m	18.90 (6.48)	2.917	0.004**	7.47 (7.40)	1.010	0.315
M_b500m - R_b500m	12.83 (5.21)	2.465	0.015*	6.81 (6.38)	1.067	0.289

Unit of the mean of difference (D) and standard error (SE): *nW*∙*cm*^−2^∙*sr*^−1^. (M: Mobility-oriented measurement, R: Residence-based measurement, CC: Cross-calibrated, r???m: *In-situ* measurement using the given spatial resolution, and b???m: Zonal mean value using the given buffer zone radius).

*Significant different from 0 at the 0.05 level, 2-tailed tests.

**Significant different from 0 at the 0.01 level, 2-tailed tests.

**Table 6:** Paired-sample t-tests of differences between measured exposures to outdoor ALAN while controlling contextual settings from NTL imagery.

Pair	SSP (N = 104)			TSW (N = 104)		
	D (SE)	*t*	*p*-value	D (SE)	*t*	*p*-value
M_r010m - M_r130m	153.16 (19.69)	7.780	<0.001**	97.96 (11.16)	8.781	<0.001**
M_r130m - M_r500m	108.05 (5.46)	19.799	<0.001**	77.70 (7.38)	10.529	<0.001**
M_b065m - M_r130m	140.55 (14.10)	9.966	<0.001**	87.18 (8.88)	9.822	<0.001**
M_b250m - M_r500m	232.28 (7.58)	30.661	<0.001**	153.05 (7.78)	19.663	<0.001**
M_r010m - M_r130m_CC	20.62 (19.44)	1.061	0.291	-1.97 (11.44)	-0.173	0.863
M_b065m - M_r130m_CC	8.02 (13.98)	0.573	0.568	-12.76 (9.72)	-1.312	0.192
M_b250m - M_r500m_CC	71.44 (8.12)	8.793	<0.001**	38.87 (7.51)	5.173	<0.001**
R_r010m - R_r130m	19.54 (15.63)	1.250	0.214	-3.26 (18.69)	-0.174	0.862
R_r130m - R_r500m	91.27 (5.06)	18.030	<0.001**	61.16 (8.83)	6.924	<0.001**
R_b065m - R_r130m	83.17 (11.18)	7.442	<0.001**	52.28 (22.61)	2.312	0.023*
R_b250m - R_r500m	210.82 (6.03)	34.967	<0.001**	136.75 (6.11)	22.395	<0.001**
R_r010m - R_r130m_CC	-107.79 (15.91)	-6.773	<0.001**	-98.38 (21.12)	-4.658	<0.001**
R_b065m - R_r130m_CC	-44.16 (11.62)	-3.801	<0.001**	-42.85 (24.79)	-1.728	0.087
R_b250m - R_r500m_CC	43.06 (7.40)	5.815	<0.001**	14.95 (7.14)	2.094	0.039*

Unit of the mean of difference (D) and standard error (SE): *nW*∙*cm*^−2^∙*sr*^−1^. (M: Mobility-oriented measurement, R: Residence-based measurement, CC: Cross-calibrated, r???m: *In-situ* measurement using the given spatial resolution, and b???m: Zonal mean value using the given buffer zone radius).

*Significant different from 0 at the 0.05 level, 2-tailed tests.

**Significant different from 0 at the 0.01 level, 2-tailed tests.

**Table 7:** Paired-sample t-tests of differences between measured exposures to outdoor ALAN while controlling contextual settings of buffer zone radius.

Pair	SSP (N = 104)			TSW (N = 104)		
	D (SE)	*t*	*p*-value	D (SE)	*t*	*p*-value
M_b500m - M_b300m	-8.67 (4.04)	-2.145	0.034*	-19.01 (3.59)	-5.289	<0.001**
M_b300m - M_b100m	-11.95 (9.04)	-1.322	0.189	-11.91 (7.46)	-1.597	0.113
R_b500m - R_b300m	-2.61 (5.44)	-0.480	0.632	-18.34 (4.47)	-4.105	<0.001**
R_b300m - R_b100m	4.95 (11.23)	0.440	0.661	30.98 (9.70)	3.192	0.002**

Unit of the mean of difference (D) and standard error (SE): *nW*∙*cm*^−2^∙*sr*^−1^. (M: Mobility-oriented measurement, R: Residence-based measurement, CC: Cross-calibrated, r???m: *In-situ* measurement using the given spatial resolution, and b???m: Zonal mean value using the given buffer zone radius).

*Significant different from 0 at the 0.05 level, 2-tailed tests.

**Significant different from 0 at the 0.01 level, 2-tailed tests.

Multiple significant disparities can also be observed between different sensors but the results are complicated (Table 6). Because of the disparities in spectral bandwidths and spatial resolutions between different sensors, it is very easy to observe significant disparities between the measurements using different sensors, using either RBM or MOM in both SSP and TSW. Cross-calibration can mitigate the disparities induced by the sensor’s spectral configurations in most cases (indicated by the smaller absolute t values), but significant disparities can still be observed. The results particularly emphasize that the disparities between different sensors and NTL images cannot be easily and adequately mitigated, and the proper delineation of urban nightscape for outdoor ALAN exposure measurement may rely on the initial proper choice of the appropriate remote sensing data source. Finally, the buffer zone size may also induce significant disparities in the outdoor ALAN exposure measurements (Table 7). Because the *in-situ* measurements using fine-grained NTL images (e.g., SDGSAT-1 10 m) may not adequately capture all causally relevant outdoor ALAN exposure, a buffer zone may be necessary in such case. However, because the nightscape in urban areas change drastically across space, the results can be vastly different when using different buffer zone radii. We observed multiple disparities due to different buffer zone sizes, but it is very difficult to summarize a consistent trend of the buffer zone’s effects either on the purely fine-grained measurements (Table 7) or on the mitigation of disparities induced by sensor configurations (Table 6).

### The modeled overall health outcomes using measured exposure to outdoor ALAN

**Fig 7:**
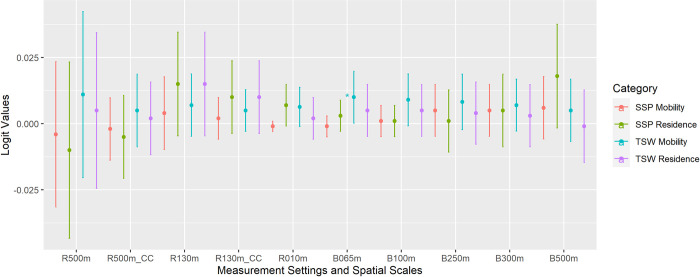
The logit values bounded with 95% confidence intervals using the designated 40 groups of outdoor ALAN exposure measurements with different contextual settings and binary logistic regression. The estimated logit values were adjusted by age, gender, educational level, marital status, and socio-economic status. r???m: *In-situ* measurements using the given spatial resolution; CC: Cross-calibrated; b???m: Zonal mean value using the given buffer radius. *Significantly different from 0 at the 0.05 level.

Improper contextual settings in the outdoor ALAN measurements may include too many contextual errors and may thus lead to erroneous conclusions and interpretations when predicting people’s health outcomes. To articulate this issue, we used our 40 groups of designated outdoor ALAN exposure measurements and logistic regression to predict participants’ self-reported overall health (Fig 7). For most cases, we observed a positive effect of exposure to outdoor ALAN on people’s overall health. Since ALAN may provide urban residents with bright space for nighttime exercises [7], may increase residents’ perception of neighborhood safety [8], and the beautiful nightscape may help release people’s mental stress [9], it is reasonable to observe a positive effect of ALAN on participants’ overall health outcomes through potential indirect causal pathways. However, we only observed one effect size of outdoor ALAN exposure in TSW that is significantly different from 0 at the 0.05 level. The corresponding measurement is derived using MOM, SDGSAT-1 10 m spatial resolution, and a 65 m buffer zone. These results indicate that it is very difficult to have a precise and causally relevant measurement of people’s exposure to outdoor ALAN. Consequentially, according to our results, we suggest the mobility-oriented measurements of people’s exposure to outdoor ALAN using fine spatial resolution and a very small buffer zone.

## Discussions

### Methodological issues when measuring people’s exposure to outdoor ALAN

Multiple significant disparities have been observed in our systematic investigation of measuring people’s exposure to outdoor ALAN, which manifests the UGCoP [25]. These disparities can be attributed to contextual errors, and these contextual errors stem from mainly two sources. The most complicated source of contextual errors is the sensors’ spectral band ranges and spatial resolutions. For example, VIIRS imagery has a spatial resolution of 500 m, and each digital number (DN) value on the imagery can be considered as the average luminosity of a circular region with a diameter of about 500 m. It is not reasonable to assume that a person can get exposed to all the light in that region at a single moment. Consequentially, the *in-situ* measurements using VIIRS imagery must contain irrelevant outdoor ALAN. Meanwhile, the spectral configuration of VIIRS may also exclude some light that is relevant to human health concerns, especially the shortwave (blue) light (Table 1). Our results indicate that sensors’ configurations have a critical influence on the delineation of people’s exposure to outdoor ALAN, while the cross-calibration between sensors and the buffer zones that are designed to mitigate the disparities between spatial resolutions cannot effectively mitigate the disparities between sensors. Correspondingly, we suggest choosing the proper remote sensing data source at the beginning. Otherwise, it is difficult to rectify the results.

The other essential disparity is between RBM and MOM of people’s exposure to outdoor ALAN, while ignoring people’s nighttime mobility is the other essential source of contextual errors [1]. These contextual errors are included in the measurements, which enlarges the uncertainties when using these measurements to model people’s health concerns. The estimated effect sizes may then have shifted magnitudes and downgraded levels of significance, and we may conclude insignificant observations while they are actually significant (i.e., Type II error) [25]. Besides those significant disparities between RBM and MOM, we also observed one single significant effect size only when using the MOM of outdoor ALAN exposure to predict people’s overall health. These results indicate that it is necessary to carefully determine the approach to measuring people’s exposure to outdoor ALAN. Otherwise, the measurements may contain too many contextual errors and may lead to erroneous conclusions. Consequentially, we would like to suggest the MOM of people’s exposure to outdoor ALAN using a finer spatial resolution and a very small buffer zone.

### The implications of this study

Our systematic investigation of the contextual settings in the measurements of people’s exposure to outdoor ALAN provides multiple essential implications for a wide range of studies that examine the health effects of outdoor ALAN exposure. First, we confirmed that different contextual settings may lead to different measured exposure to outdoor ALAN, including remote sensing data sources and sensor configurations (e.g., spectral band ranges and spatial resolutions), cross-calibration or not, measurement approaches (RBM or MOM, *in-situ* observations or buffer zone average values), and geographic settings. Second, we figured out that it is very difficult to derive a causally relevant exposure to outdoor ALAN, and erroneous and misleading results may be common due to the uncertainties in the exposure measurements. Finally, we would like to suggest using MOM, SDGSAT-1 Glimmer imagery and a very small buffer zone to derive people’s outdoor ALAN exposure.

Our observations indicate the potential positive health effects of outdoor ALAN exposure on urban residents. Outdoor ALAN may provide essential bright space for nighttime physical exercises and beautiful nightscapes may promote people’s mental health [1]. These may be the reasons why we observed such positive associations. Our study is also the first implementation for the MOM of people’s exposure to outdoor ALAN, and our observations argue that this shift of research paradigm from RBM to MOM is necessary. Meanwhile, since we observed that it is easy for outdoor ALAN exposure measurements to contain contextual errors, it may be necessary to re-assess previous studies that use RBM of outdoor ALAN exposure measurements.

### The limitation of this study

Our primary limitation is the relatively small sample size. Due to the difficulties in the data collection, this pilot study only recruited about 200 participants. Although this sample size is adequate for methodological studies of measurement uncertainties, our data collection is not adequate to articulate the disparities in the outdoor ALAN exposure measurements between different socio-demographic groups.

We also need to highlight two issues of outdoor ALAN exposure measurements induced by our data collections. First, outdoor ALAN exposure measured using remote sensing data may not directly represent the eye-level received ALAN due to the angle effect [43], whereby the disturbance of human circadian rhythms and the adverse health impacts of outdoor ALAN may not be effectively captured [1]. Portable sensors with cameras may address this issue, but such an approach considerably increases the risks of violating human privacy and thus may not be permitted. Hence, our approach using remote sensing data is still the most practical approach for individual-level epidemiological investigations with large sample sets. Secondly, our cross-sectional study within a short period yielded the so-called acute exposure to ALAN, which may not support the arguments concerning the associations between chronic exposure to ALAN and the risks of other health concerns (e.g., breast cancer and obesity) [1]. A large sample size from our future longitudinal data collection may further strengthen and extend our understanding of the uncertainties induced by contextual settings, the environmental injustice between different socio-demographic groups when measuring people’s exposure to outdoor ALAN, and other concrete health concerns like sleep disorders, mental stress, and nighttime physical exercises.

## Conclusions

In this study, we systematically investigated the measurement uncertainties induced by contextual settings in the outdoor ALAN exposure measurements in different geographic contexts, including remote sensing data sources, spatial scales, and measurement approaches (residence-based or mobility-oriented measurements, *in-situ* measurements or buffer zone average values). We observed a range of significant disparities induced by different contextual settings, which empirically manifest the UGCoP. We concluded that the disparities induced by remote sensing data sources cannot be effectively mitigated and it is necessary to choose the correct data source at the very beginning. We also suggested that it is very difficult to obtain causally relevant outdoor ALAN exposure measurements when using outdoor ALAN exposure measurements to model people’s overall health. According to our results, we suggest future studies to measure people’s outdoor ALAN exposure using mobility-oriented measurements, SDGSAT-1 Glimmer imagery, and a very small buffer zone.
